# Structure-Activity Relationship of Cinnamaldehyde Analogs as Inhibitors of AI-2 Based Quorum Sensing and Their Effect on Virulence of *Vibrio* spp

**DOI:** 10.1371/journal.pone.0016084

**Published:** 2011-01-13

**Authors:** Gilles Brackman, Shari Celen, Ulrik Hillaert, Serge Van Calenbergh, Paul Cos, Louis Maes, Hans J. Nelis, Tom Coenye

**Affiliations:** 1 Laboratory of Pharmaceutical Microbiology, Ghent University, Ghent, Belgium; 2 Laboratory of Medicinal Chemistry, Ghent University, Ghent, Belgium; 3 Laboratory for Microbiology, Parasitology and Hygiene, University of Antwerp, Antwerp, Belgium; Indian Institute of Science, India

## Abstract

**Background:**

Many bacteria, including *Vibrio* spp., regulate virulence gene expression in a cell-density dependent way through a communication process termed quorum sensing (QS). Hence, interfering with QS could be a valuable novel antipathogenic strategy. Cinnamaldehyde has previously been shown to inhibit QS-regulated virulence by decreasing the DNA-binding ability of the QS response regulator LuxR. However, little is known about the structure-activity relationship of cinnamaldehyde analogs.

**Methodology/Principal Findings:**

By evaluating the QS inhibitory activity of a series of cinnamaldehyde analogs, structural elements critical for autoinducer-2 QS inhibition were identified. These include an α,β unsaturated acyl group capable of reacting as Michael acceptor connected to a hydrophobic moiety and a partially negative charge. The most active cinnamaldehyde analogs were found to affect the starvation response, biofilm formation, pigment production and protease production in *Vibrio* spp *in vitro*, while exhibiting low cytotoxicity. In addition, these compounds significantly increased the survival of the nematode *Caenorhabditis elegans* infected with *Vibrio anguillarum*, *Vibrio harveyi* and *Vibrio vulnificus*.

**Conclusions/Significance:**

Several new and more active cinnamaldehyde analogs were discovered and they were shown to affect *Vibrio* spp. virulence factor production *in vitro* and *in vivo*. Although ligands for LuxR have not been identified so far, the nature of different cinnamaldehyde analogs and their effect on the DNA binding ability of LuxR suggest that these compounds act as LuxR-ligands.

## Introduction

Many bacteria, including *Vibrio* spp., regulate gene expression in a cell-density dependent way through a communication process termed quorum sensing (QS). In *Vibrio* spp. QS is mediated by three types of synergistically acting signalling molecules: acyl-homoserine lactones (AHL), cholera-autoinducer-1 (CAI-1) and a mixture of interconvertible molecules collectively called autoinducer-2 (AI-2) [1]–[4]. The key enzymes in the production of these molecules are LuxN, LuxS and CqsA for AHL, AI-2 and CAI-1, respectively [4]. In response to binding of the signalling molecules to their cognate receptor, a phosphorelay cascade is induced. At low population density only basal amounts of diffusible signal molecules are produced, and in this situation the receptor will act as a kinase, resulting in the phosphorylation of the downstream response regulator LuxO through a cascade involving LuxU [5]. Phosphorylation activates LuxO, resulting in the production of small regulatory RNAs [6]–[7]. These small RNAs, together with the chaperone protein Hfq, destabilize mRNA encoding the response regulator LuxR. However, when population density is sufficiently high, signalling molecules will bind to their cognate receptor and the latter will act as phosphatase, leading to a dephosphorylation of LuxO [7]. Since unphosphorylated LuxO is inactive, no small regulatory RNAs will be formed and the LuxR mRNA remains stable, resulting in the production of LuxR and ultimately an altered gene expression pattern. The virulence of several *Vibrio* spp. was previously found to be controlled by multiple QS systems making QS inhibition an interesting antipathogenic strategy [8]–[10].

Cinnamaldehyde is known to affect AI-2 QS [10], [11] and we have previously shown that cinnamaldehyde disrupts QS-regulated virulence in *Vibrio* spp. by decreasing the DNA-binding activity of the response regulator LuxR [10]. However, the exact structural elements required for QS inhibitory activity remain unclear. The development of new antipathogenic agents based on cinnamaldehyde requires the understanding of the structural reason for LuxR inhibition. To address this, a small library of cinnamaldehyde analogs was screened for their inhibitory effect on QS in *Vibrio* spp. The structural elements required for QS inhibition were identified and a mechanism of action is proposed. The effect of selected cinnamaldehyde analogs on *Vibrio* spp. virulence was evaluated *in vitro* and *in vivo* in a *Caenorhabditis elegans* assay.

## Results and Discussion

### Cinnamaldehyde and cinnamaldehyde analogs do not affect bacterial growth or bioluminescence

**Figure 1:**
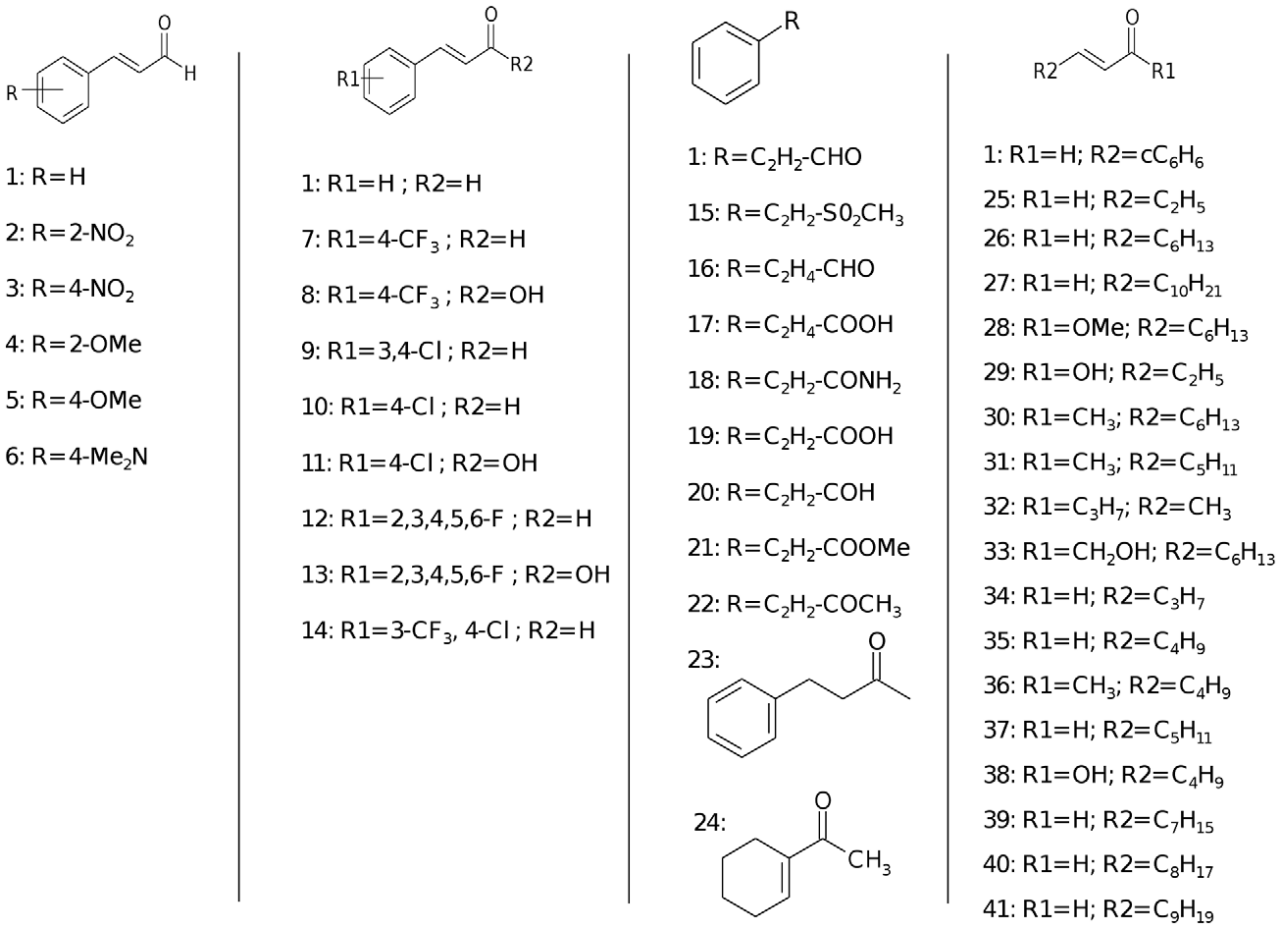
Cinnamaldehyde and cinnamaldehyde analogs used in the present study.

When used in concentrations up to 250 µM, cinnamaldehyde and most analogs (Fig. 1) did not affect the growth of the different *Vibrio* strains used in this study, the exception being 3,4-dichloro-cinnamaldehyde and 4-nitro-cinnamaldehyde (MIC ≥100 µM and MIC ≥50 µM, respectively) (data not shown). In all experiments, compounds were used in concentrations below the minimal inhibitory concentration. To rule out direct interference with bioluminescence, all compounds were assessed for their effect on the bioluminescence of an *E. coli* DH5α pBluelux strain containing the *luxCDABE* genes, but none of the compounds directly affected bioluminescence.

### Several cinnamaldehyde analogs affect AI-2-regulated bioluminescence

To screen for AI-2 inhibition, the effect of all compounds on bioluminescence of *V. harveyi* BB170 was assessed (Table 1). Five cinnamaldehyde analogs were previously shown to affect AI-2 QS. Two of these non-halogen substituted cinnamaldehyde analogs, i.e. 2-nitro-cinnamaldehyde (2) and 4-nitro-cinnamaldehyde (3), were at least as active in blocking AI-2 QS as the unsubstituted cinnamaldehyde (1) [10]. In the present study, several halogenated compounds were found to be more active than the unsubstituted cinnamaldehyde. These include 3,4-dichloro-cinnamaldehyde (9), 2,3,4,5,6-pentafluoro-cinnamaldehyde (12) and 4-chloro-3-trifluoromethyl-cinnamaldehyde (14). 3,4-Dichloro-cinnamaldehyde (9) reduced the QS-regulated bioluminescence by 99±1% without interfering with the bacterial growth of *V. harveyi* BB170. None of the halogenated cinnamic acid analogs resulted in an increased QS inhibition compared to the corresponding cinnamaldehyde analog or to the unsubstituted cinnamaldehyde. Methyl-styryl sulfone (15), cinnamamide (18) and *(E)*-4-phenyl-3-buten-2-one (22) (50 µM) resulted in a 30±11%, 28±9% and 35±16% inhibition. In addition, *(E)*-4-phenyl-2-butanone (23) and 1-acetyl-1-cyclohexene (24) were only active at higher concentrations (100 µM) while no QS inhibitory activity was observed for 3-phenylpropionaldehyde (16), 3-phenylpropionic acid (17), cinnamyl alcohol (20) and methyl cinnamate (21). Cinnamaldehyde analogs in which the aromatic ring was replaced by an alkyl moiety, but which still contain the acrolein group, proved also to be active AI-2 QS inhibitors. *(E)*-2-Pentenal (25), *(E)*-2-tridecenal (27), methyl-*(E)*-2-nonenoate (28) and *(E)*-2-heptenal (35) were at least as active in inhibiting AI-2 QS as cinnamaldehyde. *(E)*-2-Nonenal (26), *(E)-*3-decen-2-one (30), *(E)*-3-nonen-2-one (31), *(E)*-3-octen-2-one (36), *(E)*-2-octenal (37), *(E)*-2-decenal (39), *(E)*-2-undecenal (40) and *(E)*-2-dodecenal (41) led to a more pronounced inhibition of bioluminescence compared to cinnamaldehyde (1). When assayed at a higher concentration (100 µM), *(E)*-2-nonenal (26), *(E)*-3-decen-2-one (30) and *(E)*-3-nonen-2-one (31) almost completely inhibited bioluminescence. *(E)*-2-nonen-1-ol (33), *(E)*-2-hexen-1-al (34) and 2-octenoic acid (38) were less active inhibitors of AI-2 regulated bioluminescence, while 2-pentenoic acid (29) and 5-methyl-2-hepten-4-one (32) had no effect. Based on these results, cinnamaldehyde (1), 2-nitro-cinnamaldehyde (2), 3,4-dichloro-cinnamaldehyde (9), *(E)*-4-phenyl-3-buten-2-one (22), *(E)*-3-decen-2-one (30), *(E)*-2-pentenal (25) and *(E)*-2-nonenal (26) were selected for further experiments.

**Table 1:** Inhibition of QS-regulated bioluminescence in *V. harveyi* BB170 (activity is expressed as the % inhibition of the bioluminescence signal of the untreated control ± standard deviation; n≥48).

Code*	Compound	Reduction in bioluminescence (% inhibition compared to the untreated control ± SD) when used in the following concentrations:	
		*50* *µM*	*100* *µM*
1	Cinnamaldehyde**	22±4	65±13
2	2-Nitro-cinnamaldehyde**	25±5	62±7
3	4-Nitro-cinnamaldehyde**	33±7	ND
4	2-Methoxy-cinnamaldehyde**	NS	14±6
5	4-Methoxy-cinnamaldehyde**	16±4	34±9
6	4-Dimethylamino-cinnamaldehyde**	NS	17±1
7	4-Trifluoromethyl-cinnamaldehyde	19±8	21±7
8	4-Trifluoromethyl cinnamic acid	8±1	11±1
9	3,4-Dichloro-cinnamaldehyde	47±7	99±1
10	4-Chloro-cinnamaldehyde	27±2	78±5
11	4-Chloro-cinnamic acid	14±16	20±14
12	2,3,4,5,6-Pentafluoro-cinnamaldehyde	44±14	95±3
13	2,3,4,5,6-Pentafluoro-cinnamic acid	33±6	41±8
14	4-Chloro-3-trifluoromethyl-cinnamaldehyde	45±21	97±2
15	Methyl-styryl sulfone	30±11	73±6
16	3-Phenylpropionaldehyde	NS	NS
17	3-Phenylpropionic acid	NS	NS
18	Cinnamamide	28±9	61±20
19	Cinnamic acid	NS	25±20
20	Cinnamyl alcohol	NS	NS
21	Methyl cinnamate	NS	NS
22	*(E)*-4-Phenyl-3-buten-2-one	35±16	78±9
23	4-Phenyl-2-butanone	NS	13±4
24	1-Acetyl-1-cyclohexene	NS	16±6
25	*(E)*-2-Pentenal	34±12	58±22
26	*(E)*-2-Nonenal	63±3	98±2
27	*(E)*-2-Tridecenal	36±19	56±21
28	Methyl-*(E)*-2-nonenoate	32±5	59±8
29	2-Pentenoic acid	NS	NS
30	*(E)*-3-Decen-2-one	80±11	99±1
31	*(E)*-3-Nonen-2one	43±5	99±1
32	5-Methyl-2-hepten-4-one	NS	NS
33	*(E)*-2-Nonen-1-ol	NS	17±9
34	*(E)*-2-Hexen-1-al	18±8	35±3
35	*(E)*-2-Heptenal	28±6	55±1
36	*(E)*-3-Octen-2-one	48±1	80±3
37	*(E)*-2-Octenal	41±6	71±4
38	2-Octenoic acid	27±8	36±1
39	*(E)*-2-Decenal	57±6	90±6
40	*(E)*-2-Undecenal	75±9	94±6
41	*(E)*-2-Dodecenal	71±3	87±11

* ;Code refers to structures in Figure 1.

** ; previously assessed for their effect on AI-2 quorum sensing [10].

ND: not determined due to growth inhibition when used at this concentration.

NS: compound did not result in a significant inhibition of the bioluminescence signal (p>0.05; independent sample t-test).

### Cinnamaldehyde and cinnamaldehyde analogs decrease the DNA-binding ability of LuxR

**Figure 2:**
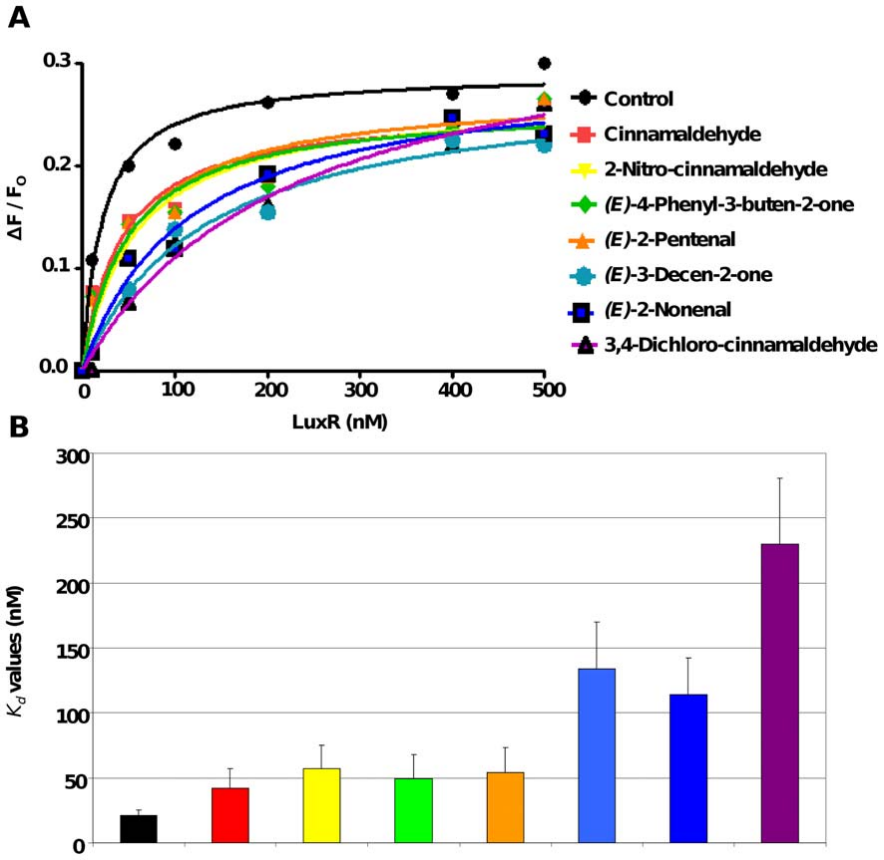
LuxR DNA binding and dissociation constants. A. DNA binding of LuxR in the absence and presence of cinnamaldehyde or a cinnamaldehyde analog (50 µM). The fractional change in anisotropy, ΔF/F_o_, is plotted against the concentration of LuxR (nM). B. *K_d_* values are calculated as the half-maximal fractional change in fluorescence anisotropy in the absence and presence of cinnamaldehyde or cinnamaldehyde analogs.

It was previously shown that cinnamaldehyde inhibits AI-2 QS by decreasing the DNA-binding ability of LuxR to its promoter DNA [10]. To assess whether the analogs also target LuxR and have the same mechanism of action, the effect of the selected compounds on bioluminescence was determined in various *V. harveyi* QS mutants (Table 2). The selected compounds were found to inhibit bioluminescence in all mutants tested, indicating that the target of these compounds is the downstream transcriptional regulatory protein LuxR (data not shown). To further investigate their effect on the DNA binding ability of LuxR, a fluorescently labelled fragment of a *V. harveyi* consensus LuxR binding sequence [12] was incubated together with purified LuxR protein in the presence and absence of cinnamaldehyde analogs. Incubation of LuxR with this DNA fragment in the absence of QS inhibitors resulted in a significant increase in anisotropy (Fig. 2). When LuxR was incubated with this DNA fragment in the presence of cinnamaldehyde, 2-nitro-cinnamaldehyde, *(E)*-4-phenyl-3-buten-2-one, *(E)*-2-pentenal, *(E)*-3-decen-2-one, *(E)*-2-nonenal or 3,4-dichloro-cinnamaldehyde, binding to DNA was strongly inhibited (Fig. 2), indicating that these compounds inhibit AI-2 mediated QS by decreasing the DNA-binding ability of LuxR.

**Table 2:** *Vibrio* strains used in this study.

Strain	Characteristics	Reference/source
*V. harveyi* BB 120	Wild-type	[2]
*V. harveyi* BB152	*luxLM*::Tn5	[6]
*V. harveyi* BB 170	*luxN*::Tn5	[1]
*V. harveyi* MM 30	*luxS*::Tn5	[30]
*V. harveyi* BB886	*luxPQ*::Tn5 Kan^R^	[6]
*V. harveyi* JAF 375	*luxN*::Cm^R^ *luxQ*::Kan^R^	[7]
*V. harveyi* JMH 597	*luxN*::Tn5 *cqsS*::Cm^R^	[9]
*V. harveyi* JAF 553	*luxU* H58A linked to Kan^R^	[5]
*V. harveyi* JAF 483	*luxO* D47A linked to Kan^R^	[7]
*V. harveyi* BNL 258	*hfq*::Tn5*lacZ*	[31]
*V. anguillarum* LMG 4411	Isolated from young sea trout (*Salmo trutta*)	BCCM/LMG
*V. cholerae* NCTC 8457	Isolated from human, biotype El Tor	HPACC
*V. cholerae* CIP 106855	Isolated from human, biotype El Tor, HapR frameshift	CIP
*V. vulnificus* LMG 16867	Isolated from tank water on eel farm	BCCM/LMG

BCCM/LMG, Belgian Co-ordinated Collections/Laboratory of Microbiology collection (Ghent University, Belgium); HPACC, Health Protection Agency Culture Collection (Salisbury, UK); CIP, Collection of Institute Pasteur (Paris, France).

### Cinnamaldehyde and cinnamaldehyde analogs affect the *in vitro* production of QS-regulated virulence factors

Subsequently we evaluated the effect of the selected compounds on the *in vitro* production of putative virulence factors. All compounds investigated were found to decrease protease activity in *Vibrio anguillarum* LMG 4411 and *Vibrio cholerae* NCTC 8457 with 25–74% (Table 3). No effects were observed on protease production in *V. cholerae* CIP 106855, a strain containing a non-functional HapR (LuxR homolog). All compounds were found to decrease the pigment production in *V. anguillarum* LMG 4411 with 15% to 65% (Table 3). This is in agreement with the previous finding that pigment production in *V. anguillarum* and protease production in *V. anguillarum* and *V. cholerae* are at least partially controlled by the AI-2 QS system [8], [10], [13].

**Table 3:** Effect of the cinnamaldehyde analogs (100 µM) on different QS-regulated phenotypes.

Code*	Compound	Protease activity		Pigment production
		*V. anguillarum*	*V. cholerae*	*V. anguillarum*
		LMG 4411	NCTC 8457	LMG 4411
1	Cinnamaldehyde	27±3	28±10	26±3
2	2-Nitro-cinnamaldehyde	39±13	27±8	36±1
9	3,4-Dichloro-cinnamaldehyde**	47±17	62±12	28±5
22	*(E)*-4-Phenyl-3-buten-2-one	43±12	32±6	25±5
30	*(E)*-3-Decen-2-one	74±15	43±8	64±20
25	*(E)*-2-Pentenal	46±6	25±9	15±7
26	*(E)*-2-Nonenal	74±17	41±7	48±8

		Biofilm biomass		
		*V. anguillarum*	*V. cholerae*	*V. anguillarum*
		LMG 4411	NCTC 8457	LMG 4411
1	Cinnamaldehyde	24±11	17±14***	21±12
2	2-Nitro-cinnamaldehyde	31±12	16 ±12***	NS
9	3,4-Dichloro-cinnamaldehyde**	16±4	27±12***	50±27
22	*(E)*-4-Phenyl-3-buten-2-one	34±9	18±11***	14±9
30	*(E)*-3-Decen-2-one	19±12	31±18***	24±14
25	*(E)*-2-Pentenal	19±11	NS	NS
26	*(E)*-2-Nonenal	23±5	29±17***	38±8

* ;Code refers to structures in Figure 1.

Results are expressed as the percentage inhibition compared to the untreated control (± SD).

NS: no significant inhibition compared to an untreated control (p>0.05; Mann-Whitney U).

** ; compound was used in 50 µM concentration.

*** ; for *V. cholerae* NCTC 8457 results are expressed as the percentage increase (instead of decrease) in biofilm biomass compared to the untreated control.

No effect was observed on *V. cholerae* CIP 106855 and *V. harveyi* BB120 biomass (data not shown), but several compounds decreased biofilm formation in *V. anguillarum* LMG 4411 and *V. vulnificus* LMG 16867 (Table 3). However, when using a resazurin-based viability assay no differences in the number of metabolically active cells in the *V. harveyi* BB120, *V. anguillarum* LMG 4411, *V. vulnificus* LMG 16867 and *V. cholerae* CIP 106855 biofilm were observed (data not shown). Mutations in the LuxR homologues of *V. anguillarum* (VanT) and *V. vulnificus* (SmcR) have been shown to reduce biofilm formation, suggesting that in these species AI-2 QS promotes biofilm formation [8], [14]. Our data support the hypothesis that cinnamaldehyde and selected cinnamaldehyde analogs affect biofilm formation by inhibiting matrix production and/or accumulation since a decrease in biomass could not be attributed to a decrease in the number of viable cells [10]. In contrast, a higher biomass was found for *V. cholerae* NCTC 8457 when treated with cinnamaldehyde or cinnamaldehyde analogs (Table 3). In addition, a significantly higher number of metabolically active cells was found in this biofilm when treated with *(E)*-2-nonenal and *(E)*-3-decen-2-one (127±14% and 114±9%, respectively). *V. cholerae* HapR has been shown to repress the expression of *vps* genes (involved in the production of exopolysaccharides) and biofilm formation, indicating that AI-2 QS negatively influences biofilm formation in this species [15], [16]. This is consistent with the observed positive effects in this study of AI-2 QS inhibitors on biofilm formation of *V. cholerae* NCTC 8457, while no effect was observed for a strain lacking a functional HapR.

The effect of the selected compounds on the starvation response of the different *Vibrio* spp. was also investigated. In the control experiments no decrease in the number of culturable cells was observed after 48 h of incubation (data not shown). However when cells were starved in the presence of one of the seven compounds tested, significantly less cells were recovered (Table 4). The highest reductions in the number of recovered cells was observed with the most active QS inhibitors, *(E)*-3-decen-2-one, 3,4-dichloro-cinnamaldehyde and *(E)*-2-nonenal (Table 4). Our data indicate that inhibition of AI-2 based QS suppresses the starvation response and renders cells more susceptible to starvation-associated stress conditions. A correlation was observed between the AI-2 QS inhibitory effect of the compounds and their effects on *in vitro* production of virulence factors.

**Table 4:** Effect of the QS inhibitors on the QS regulated starvation response (data are presented as average log reduction in CFU/ml after 48 h compared to an untreated control).

Code*	Compounds	Average log reduction CFU/ml (± SD)			
		*V. anguillarum*	*V. cholerae*	*V. harveyi*	*V. vulnificus*
		LMG 4411	NCTC 8457	BB120	LMG 16867
1	Cinnamaldehyde	1.15±0.56	NS	NS	NS
2	2-Nitro-cinnamaldehyde	1.14±0.32	1.33±0.22	NS	NS
9	3,4-Dichloro-cinnamaldehyde	>6.60***	>6.83***	5.99±0.66	3.88±0.40
22	*(E)*-4-Phenyl-3-buten-2-one	0.81±0.25	NS	NS	NS
30	*(E)*-3-Decen-2-one	2.76±0.44	3.29±0.27	5.34±0.41	1.48±0.17
25	*(E)*-2-Pentenal	1.08±0.16	1.35±0.16	2.40±0.78	NS
26	*(E)*-2-Nonenal**	>6.60***	2.64±0.54	>6.94***	2.46±0.48

* ;Code refers to structures in Figure 1.

Compounds were used at 100 µM concentrations, except 3,4-dichloro-cinnamaldehyde (50 µM).

NS: not significantly different from an untreated control (p>0.05; Mann-Whitney U).

** ; 50 µM was used for *V. cholerae* NCTC 8457.

*** ; No recovery of cells after 48 h (i.e. <20 cells/ml survived starvation).

### Cinnamaldehyde and cinnamaldehyde analogs affect virulence of *Vibrio* spp. in *C. elegans*

**Figure 3:**
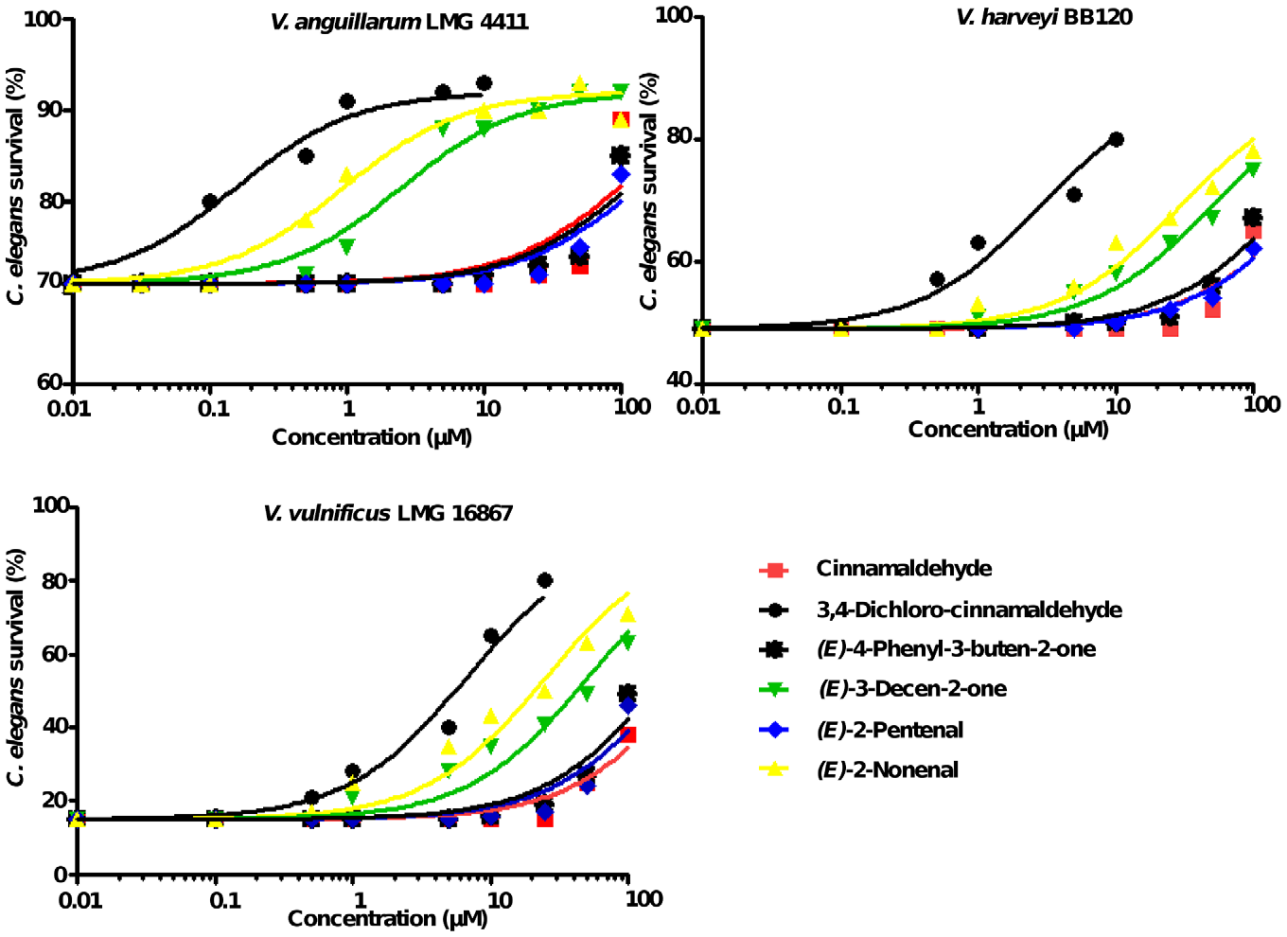
Survival curves after infection of *C. elegans* with *V. anguillarum* LMG 4411, *V. harveyi* BB120 or *V. vulnificus* LMG 16867 in the presence or absence of QS inhibitors.

To investigate the effect of the different compounds on virulence *in vivo*, *C. elegans* nematodes were infected with various *Vibrio* strains in the presence and absence of selected cinnamaldehyde analogs. Five selected compounds were found to be non-toxic towards *C. elegans* when used at 100 µM. 3,4-Di-chloro-cinnamaldehyde and 2-nitro-cinnamaldehyde were toxic in concentrations above 25 µM and, hence were used in concentrations below this threshold. In the absence of QS inhibitors, only 71±4%, 49±13% and 15±13% of the nematodes infected with *V. anguillarum* LMG 4411, *V. harveyi* BB120 and *V. vulnificus* LMG 16867 respectively, survived 48 h post infection. All QS inhibitors significantly increased survival after infection, with the most pronounced effect observed for 3,4-dichloro-cinnamaldehyde (Fig. 3). No differences in survival were observed when *C. elegans* was treated with the different compounds after infection with *V. cholerae* NCTC 8457 (data not shown).

### Structure-activity relationship of cinnamaldehyde and cinnamaldehyde analogs suggest that a Michael-type addition to LuxR residues is important for activity

Based on our observations, we hypothesized that cinnamaldehyde binds as a ligand to LuxR, thereby changing the latters conformation and consequently decreasing its ability to bind to DNA. α,β unsaturated carbonyl compounds can react with nucleophiles, in a Michael-type addition reaction. In this way, nucleophilic amino acid side chains (e.g. the thiol groups of cysteine residues) in LuxR possibly react with the electrophilic β-position to form irreversible cinnamaldehyde-receptor conjugates. This would yield a modified protein, likely resulting in a reduced ability to bind to DNA. Compounds capable of participating in a Michael-type addition reaction were found to be active, while replacement of the aldehyde group by a carboxylic acid moiety resulted in less active compounds. Compounds lacking the double bond were found to be inactive (e.g. 3-phenylpropionaldehyde and 4-phenyl-2-butanone) (Fig. 1; Table 1). Vinyl sulfone (e.g. methyl-styryl sulfone) polarizes the double bond more than the corresponding vinyl aldehyde, and thus it is no surprise that replacement of the vinyl aldehyde function by a vinyl sulfone leads to a more active compound. These data strongly suggest the involvement of a Michael-type addition reaction. The QS inhibitory effect of cinnamaldehyde analogs was also highly dependent on the nature and degree of substitution of the aromatic ring. In general, substituents with electron withdrawing properties increased activity. Examination of the physiochemical properties including electronic (Hammett sigma constant, σ) and lipophilic properties (the Hansch constant, π) further indicated that substitutions yielding higher π+σ values resulted in increased inhibition. The inhibitory activity decreased in the order 3,4-dichloro-cinnamaldehyde (9)/4-chloro-3-trifluoromethyl-cinnamaldehyde (14) >4-chloro-cinnamaldehyde (10) > cinnamaldehyde (1). Although 4-trifluoromethyl-cinnamaldehyde (7) has the same σ value as 3,4-dichloro-cinnamaldehyde (9), its lower inhibitory activity is likely due to its lower hydrophobicity and the lack of a meta-sterical effect [17]-[18]. In addition, 4-nitro-cinnamaldehyde (3) has a lower activity than 3,4-dichloro-cinnamaldehyde (9) indicating that a favourable σ value can be neutralised by an unfavourable π effect.

Recently, the crystal structure of two LuxR homologues, SmcR in *V. vulnificus* and HapR in *V. cholerae*, was described [19]–[20]. Both proteins show conserved putative ligand-binding sites which are surrounded by polar amino acids side chains on one side of the pocket and by hydrophobic residues on the other side [19]. Further analysis revealed a positive charge in the pocket [20]. These results indicate that for optimal binding affinity a ligand should contain a hydrophobic side chain as well as a (partially) negative charge. Although no small molecule ligands which fit these pockets have been identified so far, the amphipatic nature of cinnamaldehyde suggests that it might act as a LuxR-ligand, thereby changing the DNA-binding ability of LuxR. However, in order to gather additional support for this proposed mechanism and in order to identify the site(s) of modification in LuxR by cinnamaldehyde or cinnamaldehyde analogs, the protein-inhibitor complex should be investigated. Unfortunately, the currently available overexpression construct (GST-LuxR) [12] does not allow to purify LuxR in sufficient quantities to permit analyzing the protein-inhibitor complex by X-ray crystallography or by MALDI-MS (data not shown).

### The active AI-2 inhibiting cinnamaldehyde analogs have drug-like properties

In order to be potentially useful as therapeutic agents, active molecules need to satisfy several criteria. The topological polar surface area (TPSA) is, together with the molecular volume, lipophilicity and solubility, widely acknowledged as an important factor determining transport of drugs across membranes. It has been suggested that passively absorbed compounds should have a maximum TPSA of 120 Å^2^. In addition, there is reasonable probability that compounds are well absorbed when they have logP (octanol/water partition coefficient) values below 5.0. All compounds used have a TPSA below 120 Å^2^ (Supplementary Table S1). In addition, the most active AI-2 QS inhibitors all have a logP value above 2 and a molecular volume of 130–150 Å^3^ (Supplementary Table S1). All compounds were further evaluated for compliance with Lipinski's ‘rule-of-five’ [21]. This rule summarizes important molecular pharmacokinetic properties of a compound that make it potentially applicable as an oral drug. Most drug-like molecules have logP <5, molecular weight <500 and should contain not more than 10 hydrogen bond acceptors and less than 5 hydrogen bond donors. None of the most active QS inhibiting compounds violated this ‘rule-of-five’. However, these compounds are likely to react irreversibly with different proteins, a property that is preferably avoided in drug development.

The cytotoxicity of the most promising compounds, 3,4-dichloro-cinnamaldehyde (9), *(E)*-2-nonenal (26) and *(E)*-3-decen-2-one (30) was evaluated against MRC-5 cells. An IC_50_ value of 22±1 µM was found for 3,4-dichloro-cinnamaldehyde (9). This result is in agreement with the observed toxicity of this compound towards *C. elegans*. In contrast, IC_50_ values for cinnamaldehyde (1), *(E)*-3-decen-2-one (30) and *(E)*-2-nonenal (26) were much higher (77±22 µM, >640 µM and 192±97 µM, respectively). In addition, *(E)*-2-nonenal (26) and *(E)*-3-decen-2-one (30) proved active blockers of *in vivo* virulence at low micromolar concentrations (even sub-micromolar for 3,4-dichloro-cinnamaldehyde), which suggests that the therapeutic window of these compounds is high enough for therapeutic applications in humans and animals.

### Conclusion

By evaluating the effect of several cinnamaldehyde analogs on AI-2 QS, structural elements important for AI-2 QS inhibitors could be identified. These structural elements consist of an α,β unsaturated side chain capable of reacting through Michael addition, a hydrophobic moiety as well as a (partially) negative charge. Although no small molecule ligands for LuxR have been identified so far, the chemical structure of cinnamaldehyde analogs and their effect on the DNA binding ability of LuxR led us to the hypothesis that cinnamaldehyde analogs can act as LuxR-ligands, thereby changing the DNA-binding ability of LuxR. In addition, the most active cinnamaldehyde analogs were found to reduce the *Vibrio* species starvation response, to affect biofilm formation in *V. anguillarum*, *V. vulnificus* and *V. cholerae*, to reduce pigment production in *V. anguillarum* and protease production in *V. anguillarum and V. cholerae*, and to increase the survival of *C. elegans* nematodes infected with *V. anguillarum*, *V. harveyi* and *V. vulnificus.* Finally, the most promising compounds have drug-like properties and exhibited only low cytotoxicity towards a MRC-5 cell line.

## Materials and Methods

### Cinnamaldehyde and analogs

Cinnamaldehyde (1), methyl-styryl sulfone (15), 3-phenylpropionaldehyde (16), 3-phenylpropionic acid (17), cinnamamide (18), cinnamic acid (19), cinnamyl alcohol (20), methyl cinnamate (21), *(E)*-4-phenyl-3-buten-2-one (22), 4-phenyl-2-butanone (23), 1-acetyl-1-cyclohexene (24), *(E)*-2-pentenal (25), *(E)*-2-nonenal (26), *(E)*-2-tridecenal (27), methyl-*(E)*-2-nonenoate (28), 2-pentenoic acid (29), *(E)*-3-decen-2-one (30), *(E)*-3-nonen-2-one (31), 5-methyl-2-hepten-4-one (32), *(E)*-2-nonen-1-ol (33), *(E)*-2-hexen-1-al (34), *(E)*-2-heptenal (35), *(E)*-3-octen-2-one (36), *(E)*-2-octenal (37), 2-octenoic acid (38), *(E)*-2-decenal (39), *(E)*-2-undecenal (40) and *(E)*-2-dodecenal (41) were obtained from Sigma-Aldrich (Bornem, Belgium). 2-Methoxy-cinnamaldehyde (4) was obtained from Wako Pure Chemical Industries (Osaka, Japan). 4-Methoxy-cinnamaldehyde (5) was obtained from VWR International (West Chester, PA, USA) and 2-nitro-cinnamaldehyde (2), 4-nitro-cinnamaldehyde (3), 4-dimethylamino-cinnamaldehyde (6), 4-trifluoromethyl-cinnamic acid (8), 4-chloro-cinnamic acid (11) and 2,3,4,5,6-pentafluoro-cinnamic acid (13) were obtained from Acros Organics (Geel, Belgium). 4-Chloro-cinnamaldehyde (10) and 4-chloro-3-trifluoromethyl-cinnamaldehyde (14) were synthesized via a Wittig reaction as previously described [22]. The halogenated cinnamaldehyde analog 3,4-dichloro-cinnamaldehyde (9) was synthesized as previously described [23]. The halogenated cinnamaldehyde analogs 4-trifluoromethyl-cinnamaldehyde (7) and 2,3,4,5,6-pentafluoro-cinnamaldehyde (12) were synthesized by conversion of the corresponding cinnamic acid analogs into acid chlorides and subsequent reduction with lithium tri-*tert*-butoxyaluminiumhydride [24]. Stock solutions of all compounds were stored at −20°C.

### Bacterial strains, nematodes and growth media

All *Vibrio* strains (Table 2) were routinely cultured overnight in Marine Broth (MB) (BD, Sparks, MD, USA) at 30°C on a rotary shaker. *E. coli* BL21 pGET-1 (containing the *gst-luxR* overexpression construct) and *E. coli* DH5α pBlueLux (containing pBluelux polylinker and *luxCDABE* genes) were grown in Luria-Bertani broth with aeration at 37°C in the presence of ampicillin (100 µg/ml). *E. coli* OP50 was routinely cultured in TSB at 37°C.

*C. elegans* N2 (*glp-4; sek-1*) was propagated on nematode growth agar (0.25% peptone, 0.3% NaCl, 1.7% agar, 5 mg cholesterol, 1 mM CaCl_2_, 1 mM MgSO_4_, 25 mM phosphate buffer) containing 100 µg/ml kanamycin and with *E. coli* OP50 as a food source. *C. elegans* adults were harvested as previously described [25].

### Determination of the minimal inhibitory concentration (MIC)

MICs were determined for each compound and strain as described previously [26]. In brief, a microdilution assay in flat bottomed 96-well microtiter plates (TPP, Trasadingen, Switzerland), using MB as a medium was used. The plates were incubated for 24 h at 30°C and the absorption at 590 nm was measured using a Victor Wallac^2^ multilabel counter (Perkin Elmer Life and Analytical Sciences, Boston, MA, USA). In addition, MIC values were also determined for pathogens employing an AI-2 mediated QS system. These pathogens are *Escherichia coli* BW 25113, *E. coli* K12, *E. coli* LMG 25922; *Salmonella enterica* serovar Typhimurium ATCC 700720, *Staphylococcus aureus* LMG 10147 and *S. aureus* Mu50. When used in concentrations up to 1000 µM, cinnamaldehyde and most cinnamaldehyde analogs did not affect the growth of the different strains (with the exception of 3,4-dichloro-cinnamaldehyde, MIC ≥250 µM).

### Bioluminescence assays

The assay for the effect on constitutively expressed bioluminescence (using *E. coli* DH5α pBlueLux containing the *luxCDABE* genes) and the bioassay to determine the molecular target of the compounds tested (using *V. harveyi* BB120, BB152, BB170, BB886, BNL258, JAF375, JAF553, JAF483, JMH597 and MM30) were conducted as described previously [27]. Each compound was tested at least six times in triplicate (n≥18). Inhibitory effects on AI-2 QS were assessed using a *V. harveyi* BB170 assay [10]. Each compound was tested twelve times in each assay and each assay was repeated at least four times (n≥48).

### LuxR-DNA binding assay

Induction of GST-LuxR overexpression and protein purification were conducted as previously described [12]. GST-LuxR was purified using Glutathione Uniflow resins (Clontech, Mountain view, CA, USA) and fractions containing GST-LuxR were identified by both SDS-PAGE and capillary electrophoresis (Experion PRO260 chip; Bio-rad laboratories, Nazareth Eke, Belgium). 5′fluorescein-labelled DNA oligonucleotide (TATTGATAAATTTATCAATAA) and its unlabelled complement were obtained from Sigma-Aldrich. Annealing of the complementary oligonucleotides was achieved by heating equimolar concentrations in NaCl-Tris-EDTA buffer at 94°C for 2 min, after which the reaction mixtures were allowed to slowly cool to room temperature. Fluorescence polarisation measurements in the presence and absence of QS inhibitors were conducted as described previously [12]. Samples were excited at 480 nm and emission was measured at 535 nm on a Perkin Elmer EnVision plate reader at 30°C. *K*_d_ values were calculated as the concentration of LuxR at the half-maximal fractional change in fluorescence anisotropy and curves were fit by non-linear regression using the Graphpad software (Graphpad software Inc., La Jolla, CA, USA).

### Effect of cinnamaldehyde analogs on QS-regulated phenotypes *in vitro*

The effect on protease production in *V. anguillarum* LMG 4411 and *V. cholerae* NCTC 8457 was conducted using an azocasein assay. In brief, strains were grown overnight in LB at 30°C in the presence and absence of QS inhibitors and the OD at 605 nm was determined. Five-hundred microliter of cell-free supernatant was combined with 500 µl azocasein (5 mg/ml in 100 mM Tris, pH 8) and incubated for 1 h at 37°C. After incubation, 100 µl 10% trichloroacetic acid was added and the mixture was centrifuged. The supernatant was then transferred to 700 µl NaOH (525 mM) and the optical density at 420 nm was measured.

The effect on pigment production in *V. anguillarum* LMG 4411 was evaluated as described previously [8]. Each compound was tested twice in each assay and each assay was repeated at least four times (n≥8).

Biofilms were grown and biofilm biomass was quantified by crystal violet (CV) staining using a Perkin Elmer EnVision plate reader, as described previously [10], [28]. For quantification of the number of metabolically active cells in the biofilm, a resazurin assay was used [28]. Each compound was tested six times in each assay and each assay was repeated at least three times (n≥18).

The effect of the QS inhibitors on starvation response of several *Vibrio* spp. was performed as described previously [27]. Each assay was repeated at least three times.

### *C. elegans* challenge test

Synchronized adult nematode populations were obtained as described previously [29]. For the survival assay, synchronized L4 worms were suspended in a medium containing 95% M9 buffer, 5% BHI and 10 µg/ml cholesterol (Sigma-Aldrich). 0.5 ml of this suspension was transferred to the well of a 24-well plate. An overnight bacterial culture was centrifuged and resuspended in the assay medium and standardized to 10^8^ CFU/ml. 250 µl aliquots of the standardized bacterial population were added to each well, while 250 µl sterile medium was added to the positive control. QS inhibitors were added to the test-wells. The assay plates were incubated at 25°C for up to two days. The fraction of death worms was determined by counting the number of dead worms and total number of worms in each well using a dissecting microscope. Each compound was tested three times in each assay and each assay was repeated at least three times (n≥9).

### Investigation of pharmacokinetic properties and cytotoxicity

The molecular properties of all QS inhibiting compounds were predicted using a commercial software package (Molinspiration Cheminformatics, Slovensky Grob, Slovak Republic). Cytotoxicity was tested on human simian virus 40-immortalized lung fibroblasts (MRC-5 SV_2_ cells; European Collection of Cell Cultures, United Kingdom). MRC-5 cells were cultured in Earl's MEM (Gibco, UK) +5% FCSi. Assays were performed in 96-well microtiter plates, each well containing about 10^4^ cells. After 3 days incubation at 37°C with 5% CO_2_, cell viability was assessed fluorimetrically after addition of resazurin (excitation and emission wavelength of 550 nm and 590 nm, respectively). The results are expressed as % reduction in cell growth/viability compared to untreated control wells and an IC_50_ is determined.

### Statistics

The normal distribution of the data was checked using the Shapiro–Wilk test. Normally and non-normally distributed data were analyzed using an independent sample t-test and the Mann–Whitney U test, respectively. Statistical analyses were carried out using SPSS software, version 17.0 (SPSS, Chicago, IL, USA).

## Supporting Information

Table S1 The molecular properties of the different compounds.
(DOC)
