# Supplementary material for: Structure-Activity Relationship of Cinnamaldehyde Analogs as Inhibitors of AI-2 Based Quorum Sensing and Their Effect on Virulence of Vibrio spp
Source: PLoS One. 2011 Jan 13;6(1):e16084. doi: 10.1371/journal.pone.0016084 (PMC3020944; doi:10.1371/journal.pone.0016084)
Supplement: Table S1 — The molecular properties of the different compounds. (DOC) [file pone.0016084.s001.doc]

**Supplementary Table S1**. The molecular properties of the different compounds.

| **Code** | **Compounds** | ***MW (Da)*** | ***Volume (Å³)*** | ***LogP*** | ***TPSA (Å²)*** |
| --- | --- | --- | --- | --- | --- |
| 1 | Cinnamaldehyde | 132.162 | 130.444 | 2.484 | 17.071 |
| 2 | 2-Nitro-cinnamaldehyde | 177.159 | 153.778 | 2.215 | 62.895 |
| 3 | 4-Nitro-cinnamaldehyde | 177.159 | 153.778 | 2.443 | 62.895 |
| 4 | 2-Methoxy-cinnamaldehyde | 162.188 | 155.990 | 2.313 | 26.305 |
| 5 | 4-Methoxy-cinnamaldehyde | 162.188 | 155.990 | 2.54 | 26.305 |
| 6 | 4-Dimethylamino-cinnamaldehyde | 175.231 | 176.350 | 2.586 | 20.309 |
| 7 | 4-Trifluoromethyl-cinnamaldehyde | 200.159 | 161.741 | 3.379 | 17.071 |
| 8 | 4-Trifluoromethyl cinnamc acid | 216.158 | 169.759 | 2.805 | 37.299 |
| 9 | 3,4-Dichloro-cinnamaldehyde | 201.052 | 157.515 | 3.768 | 17.071 |
| 10 | 4-Chloro-cinnamaldehyde | 166.607 | 143.980 | 3.162 | 17.071 |
| 11 | 4-Chloro-cinnamic acid | 182.606 | 151.997 | 2.588 | 37.299 |
| 12 | 2,3,4,5,6-Pentafluoro-cinnamaldehyde | 222.112 | 155.100 | 2.835 | 17.071 |
| 13 | 2,3,4,5,6-Pentafluoro-cinnamic acid | 238.111 | 163.118 | 2.26 | 37.299 |
| 14 | 4-Chloro-3-trifluoromethyl-cinnamaldehyde | 234.604 | 157.277 | 3.985 | 17.071 |
| 15 | Methyl-styryl sulfone | 182.244 | 159.453 | 1.722 | 34.142 |
| 16 | 3-Phenylpropionaldehyde | 134.178 | 136.631 | 2.455 | 17.071 |
| 17 | 3-Phenylpropionic acid | 150.177 | 144.648 | 1.88 | 37.299 |
| 18 | Cinnamamide | 147.177 | 141.732 | 1.395 | 43.094 |
| 19 | Cinnamic acid | 148.161 | 138.462 | 1.91 | 37.299 |
| 20 | Cinnamyl alcohol | 134.178 | 136.280 | 2.032 | 20.228 |
| 21 | Methyl cinnamate | 162.188 | 155.990 | 2.526 | 26.305 |
| 22 | *(E)*-4-Phenyl-3-buten-2-one | 146.189 | 147.005 | 2.209 | 17.071 |
| 23 | 4-Phenyl-2-butanone | 148.205 | 153.192 | 2.18 | 17.071 |
| 24 | 1-Acetyl-1-cyclohexene | 124.183 | 131.962 | 1.966 | 17.071 |
| 25 | *(E)*-2-Pentenal | 84.118 | 92.398 | 1.530 | 17.071 |
| 26 | *(E)-*2-Nonenal | 140.226 | 159.605 | 3.843 | 17.071 |
| 27 | *(E)*-2-Tridecenal | 196.334 | 226.812 | 5.863 | 17.071 |
| 28 | Methyl-*(E)*-2-nonenoate | 170.252 | 185.151 | 3.885 | 26.305 |
| 29 | 2-Pentenoic acid | 100.117 | 100.416 | 0.956 | 37.229 |
| 30 | 3-Decen-2-one | 154.253 | 176.166 | 3.568 | 17.071 |
| 31 | *(E)*-3-Nonen-2one | 140.226 | 159.364 | 3.063 | 17.071 |
| 32 | 5-Methyl-2-hepten-4-one | 126.199 | 142.348 | 2.002 | 17.071 |
| 33 | *(E)*-2-Nonen-1-ol | 142.242 | 165.441 | 3.390 | 20.228 |
| 34 | *(E)*-2-Hexen-1-al | 98.145 | 109.2 | 2.327 | 17.071 |
| 35 | *(E)*-2-Heptenal | 112.172 | 126.002 | 2.832 | 17.071 |
| 36 | *(E)*-3-Octen-2-one | 126.199 | 142.563 | 2.558 | 17.071 |
| 37 | *(E)*-2-Octenal | 126.199 | 142.803 | 3.337 | 17.071 |
| 38 | 2-Octenoic acid | 144.214 | 157.008 | 3.017 | 37.299 |
| 39 | *(E)*-2-Decenal | 154.253 | 176.407 | 4.348 | 17.071 |
| 40 | *(E)*-2-Undecenal | 168.28 | 193.209 | 4.853 | 17.071 |
| 41 | *(E)*-2-Dodecenal | 182.307 | 210.011 | 5.358 | 17.071 |
